# Factors restricting the range expansion of the invasive green anole *Anolis carolinensis* on Okinawa Island, Japan

**DOI:** 10.1002/ece3.3002

**Published:** 2017-05-10

**Authors:** Yukari Suzuki‐Ohno, Kenjiro Morita, Nobuaki Nagata, Hideaki Mori, Shintaro Abe, Takashi Makino, Masakado Kawata

**Affiliations:** ^1^ Department of Ecology and Evolutionary BiologyGraduate School of Life SciencesTohoku UniversitySendaiMiyagiJapan; ^2^ Ogasawara DivisionJapan Wildlife Research CenterTokyoJapan; ^3^ Naha Nature Conservation OfficeMinistry of the EnvironmentNahaOkinawaJapan

**Keywords:** adaptation, conservation, invasion, MaxEnt, species distribution model, time lag

## Abstract

The green anole *Anolis carolinensis* invaded the Ogasawara Islands in Japan, drove various native species to extinction, and its distribution expanded 14 years after initial establishment. *A. carolinensis* invaded Okinawa Island, but it has not expanded its distribution in more than 25 years, although its density is extremely high in the southern region. To determine whether *A. carolinensis* has the potential to expand its distribution on Okinawa Island, we performed phylogenetic analysis of mitochondrial ND2 DNA sequences to study the origin of *A. carolinensis* that invaded Okinawa Island. We further used a species distribution model (MaxEnt) based on the distribution of native populations in North America to identify ecologically suitable areas on Okinawa Island. Nucleotide sequence analysis shows that the invader *A. carolinensis* originated in the western part of the Gulf Coast and inland areas of the United States and that a portion of the anoles on Okinawa was not introduced via the Ogasawara Islands. The MaxEnt predictions indicate that most areas in Okinawa Island are suitable for *A. carolinensis*. Therefore, *A. carolinensis* may have the potential to expand its distribution in Okinawa Island. The predictions indicate that habitat suitability is high in areas of high annual mean temperature and urbanized areas. The values of precipitation in summer in the northern region of Okinawa Island were higher compared with those of North America, which reduced the habitat suitability in Okinawa Island. Adaptation to low temperatures, an increase in the mean temperature through global warming, and an increase in open environments through land development will likely expand the distribution of *A. carolinensis* in Okinawa Island. Therefore, we must continue to monitor the introduced populations and be alert to the possibility that city planning that increases open environments may cause their range to expand.

## 1 Introduction

Understanding the factors that affect the expansion and restriction of the distribution of invasive species is crucial for the conservation of native species as well as to identify evolutionary biological adaptations. Invasive species commonly show a lag time before the onset of range expansion (Cox, 2004; Kowarik, 1995; Mack, 1985; Sakai et al., 2001), which can be explained by ecological and evolutionary factors (Cox, 2004; Sakai et al., 2001). The lag may be explained by ecological factors when the population is in the initial low‐density phase of exponential growth. Evolutionary factors cause a lag through the failure to adapt to the new habitat and the genetic load owing to an initial small effective population size (Ellstrand & Schierenbeck, 2000; Mack et al., 2000). In particular, if the distribution of a successfully established species is initially restricted and cannot expand its range to a new habitat over a long time, this lag time may be required for evolutionary adaptation. Management will likely be most effective if we can identify the factors that restrict range expansion of the invasive species during the lag phase, allowing the implementation of measures to arrest the expansion.

The green anole *Anolis carolinensis* is an *Anolis* lizard, which represents a typical example of adaptive radiation (Losos, 2009). It is native to the southeastern United States and expanded its distribution to the northern and southwestern regions (Campbell‐Staton et al., 2012; Losos, 2009). Further, *A. carolinensis* was introduced to other areas, particularly Pacific Ocean islands such as Guam and Hawaii (Glor, Losos, & Larson, 2005; Lever, 2003). In 1965, *A. carolinensis* was found on Chichi‐jima Island, one of the oceanic Ogasawara (Bonin) Islands, which comprise an archipelago of over 30 islands 1,000 km south of Tokyo, Japan (Hasegawa, 1986; Matsumoto, Matsumono, & Miyashita, 1980). *A. carolinensis* was introduced to Haha‐jima Island in the Ogasawara archipelago between 1980 and 1985 (Miyashita, 1991; Suzuki & Nagoshi, 1999). The *A. carolinensis* populations on the Chichi‐jima and Haha‐jima Islands exhibited lag times of 14 and 6 years, respectively, before their populations expanded and increased. After the lag phase, *A. carolinensis* expanded its range on the Ogasawara Islands and had a significant negative impact on native species and the ecosystem. *A*. *carolinensis* consumes arboreal and diurnal insects, which caused the near extinctions of an endemic butterfly species, *Celastrina ogasawaraensis*, five endemic dragonfly species as well as large reductions in the populations of cicadas, longhorn beetles, and small bee species on Chichi‐jima and Haha‐jima Islands (Abe, Makino, & Okochi, 2008; Makihara, Kitajima, Goto, Kato, & Makino, 2004; Toda, Nakagawa, & Sukigara, 2009). Further, *A. carolinensis* competes with and preys upon an endemic lizard *Cryptoblepharus boutonii nigropunctatus* (Suzuki & Nagoshi, 1999; Toda et al., 2009). Japan has regulated the populations of *A. carolinensis* since 2005, according to the provisions of the Invasive Alien Species Act.

**Figure 1:**
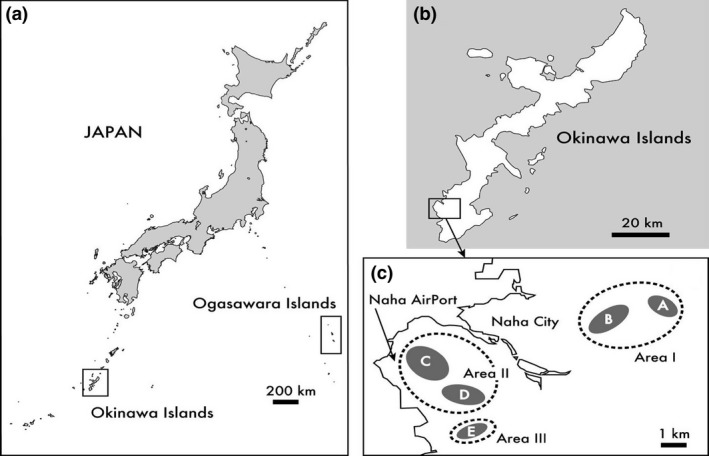
Map of Japan, including the Ogasawara Islands and Okinawa Island. (a) Okinawa Islands, (b) present distribution areas of *A. carolinensis* in Okinawa Island, (c) the three high‐density areas including five populations of *A. carolinensis* reported by the Naha Nature Conservation Office of the Ministry of the Environment Japan (2010, 2011) [Correction added on 18 May 2017 after first online publication: Figure 1 has now been replaced with an updated version.]

*Anolis. carolinensis* was first captured in 1989 on Okinawa Island, approximately 630 km south of the Japanese mainland and 1,440 km west of the Ogasawara Islands (Figure 1) (Chigira, 1991), and it did not expand its distribution until more than 25 years later. Since 2009, the Naha Nature Conservation Office of the Ministry of the Environment, Japan has conducted pilot surveys to investigate the distribution and population density of *A. carolinensis* by collecting visual observational data from citizens and by capturing the lizards using adhesive traps. These surveys show that viable *A. carolinensis* populations were established in restricted areas of Naha City and Tomigusuku City (Naha Nature Conservation Office of the Ministry of the Environment, Japan 2010, 2011, 2012). Sighting reports and interviews with residents indicate that the distribution of *A. carolinensis* was restricted to the southern region of Okinawa Island, from Tomigusuku to Ginowan (Ishikawa et al., 2012). Some *A. carolinensis* populations are now highly dense within a restricted distribution area in the southern region, although the distribution range shows no tendency for expansion. Natural forests are preserved in the northern part of Okinawa Island, and important native species inhabit these forests, including the damselfly *Matrona basilaris japonica* and the endangered Kuroiwa's ground gecko *Goniurosaurus kuroiwae kuroiwae*. An invasion of *A. carolinensis* into these areas may have a significant impact on these species through predation or competition. It is therefore important to determine whether *A. carolinensis* has the potential to expand its distribution on Okinawa Island.

The main purpose of this study was to determine whether *A. carolinensis* populations might expand their distribution on Okinawa Island. We conducted nucleotide sequence analysis of mitochondrial DNA to identify the origin of Okinawa populations and then used a species distribution model to investigate environmental factors to explain the present distribution of *A. carolinensis*. The ecologically suitable habitat of invasive species in non‐native areas is frequently predicted using a species distribution model according to their presence data in native areas (Elith & Leathwick, 2009; Herborg, Jerde, Lodge, Ruiz, & Maclsaac, 2007; Peterson, 2003). We predicted the ecologically suitable habitat of *A. carolinensis* from the presence of their populations in their native areas in North America. If the predicted suitable areas correspond to the present distribution in Okinawa Island, environmental factors may simply restrict the distribution. In contrast, if the predicted suitable areas are wider compared with their present distributions on Okinawa Island, native populations may potentially expand their distribution, although factors other than environmental variables may limit distribution. We discuss below the factors restricting the range expansion of *A. carolinensis* as well as its potential for a future expansion on Okinawa Island.

## 2 Methods

### 2.1 Sample collection

The Naha Nature Conservation Office of the Ministry of the Environment Japan (2010, 2011) reported that viable populations of *A. carolinensis* are established in five populations in three areas in Naha City and Tomigusuku City (Figure 1c). We collected 45 anole lizards in these three areas (nine from area I, 19 from area II, and 17 from area III) in 2013. We collected anoles from different regions because the populations in such different regions might include different mitochondrial haplotypes. We captured nine anoles on the Hawaiian Islands in 2014. Seven samples from Chichi‐jima Island, Ogasawara Islands were provided by the Japan Wildlife Research Center. Captured individuals were sedated, decapitated, immediately preserved in 99.9% ethanol, and then refrigerated according to the guidelines for animal care of Tohoku University.

### 2.2 DNA extraction and sequencing

Total DNA was extracted from muscle tissue of the right hind limb using a Wizard DNA extraction kit (Promega, Madison, WI, USA) according to the manufacturer's instructions. The quality and quantity of extracted DNA were evaluated using a NanoDrop 1500 (Thermo Scientific, Wilmington, DE, USA). The primers used for amplification and sequencing of mitochondrial ND2 were H5730 (Glor et al., 2004) and L4437 (Macey, Larson, Ananjeva, & Papenfuss, 1997). PCR products were purified using standard PEG sedimentation and then sequenced using an ABI 3130 Genetic Analyzer (Applied Biosystems, Foster City, CA, USA).

### 2.3 Phylogeny and population genetics

To determine the relationships between introduced and native populations, we constructed a Bayesian inference phylogenetic tree using MrBayes 3.2.2 (Ronquist et al., 2012). Sequences of 335 North American and two Ogasawaran *A. carolinensis* anoles (Table S1) and *A. porcatus* (accession number EU106343) as an out‐group were obtained from GenBank (Campbell‐Staton et al., 2012; Glor et al., 2005; Hayashi, Shima, & Suzuki, 2009; Kolbe et al., 2007; Krysko, MacKenzie‐Krysko, Connor, Alfonso, & Nunez, 2015; Tollis, Ausubel, Ghimire, & Boissinot, 2012). The substitution models for each codon (General Time Reversal model gamma for the first position and HKY85 gamma for the second and third positions) were selected using Kakusan4 (Tanabe, 2011) according to the BICc4 criterion. We performed ten million generations of MCMC sampling with a frequency of 1,000 and then discarded the first 25% of trees as burn‐in. The absolute number of nucleotide substitutions among each sequence was calculated using PAUP* (Swofford, 2003).

### 2.4 Prediction of suitable habitat area for *A. carolinensis*

To predict the ecologically suitable area and factors restricting the distribution of *A. carolinensis* in Okinawa Island, we used Maximum Entropy Model (MaxEnt) ver. 3.3.3k (Phillips, Anderson, & Schapire, 2006; Phillips & Dudik, 2008) to build a species distribution model of *A. carolinensis*. MaxEnt was suitable for this purpose because it requires presence‐only data and not absence data. The presence and environmental data used for MaxEnt are described below.

### 2.5 Presence and environmental data

*Anolis carolinensis* data in the Global Biodiversity Information Facility (GBIF) were used as the presence data of native populations (GBIF.org [Link]). Spatial filtering processing was performed by selecting one record within a cell of 10 km. This spatial filtering reduced the presence data of GBIF to 824 points.

To predict suitable habitat areas on Okinawa according to the presence data of North American populations, WorldClim and MODIS land‐cover data were used as climate, altitude, and land‐use data. Bioclim 1–19 (Table S2) and altitude data were downloaded from WorldClim ( http://www.worldclim.org) (Hijmans, Cameron, Parra, Jones, & Jarvis, 2005), and 16 MODIS land covers (Table S3) were downloaded from the Global Land Cover Facility ( http://www.glcf.umd.edu) (Channan, Collins, & Emanuel, 2014; Friedle et al., 2010). The 5 arc‐min (10‐km) resolution of bioclim 1–19, altitude, and land cover data was selected to match the resolution of the presence data obtained from GBIF in a training dataset. Altitude data included some erroneous values, which were redesignated “no data” using ArcGIS 10.0. The background region for the prediction was North America. We did not use a bias file that limits the background region according to sampling effort because we did not know the sampling effort of *A. carolinensis* data in GBIF. For the environmental projection layers, we used a 5 arc‐min resolution of bioclim 1–19, altitude data, and land‐cover data on Okinawa Island.

### 2.6 MaxEnt settings

To prevent over‐fitting, we restricted functional forms to linear and quadratic features (Merow, Smith, & Silander, 2013; Syfert, Smith, & Coomes, 2013; Syfet et al., 2014). Increasing the regularization multiplier is also effective in preventing data over‐fitting (Anderson & Gonzalez, 2011; Phillips et al., 2006). We ran MaxEnt with regularization multipliers 1 and 2; however, high‐suitability areas with regularization multiplier 2 were almost the same as those with regularization multiplier 1 (data not shown). Therefore, we show predictions with linear and quadratic features and a regulation multiplier 1 in the Results section. We selected 25% of presence data for testing with 100 prediction replicates: random test percentage = 25; maximum number of background points = 10,000; replicates = 100; replicated run type = subsample. The prediction accuracy was calculated as areas under receiver operating characteristic Curve (AUC). A jackknife test was used for checking the effects of environmental variables on the prediction accuracy.

## 3 Results

### 3.1 Origin of invader populations

**Figure 2:**
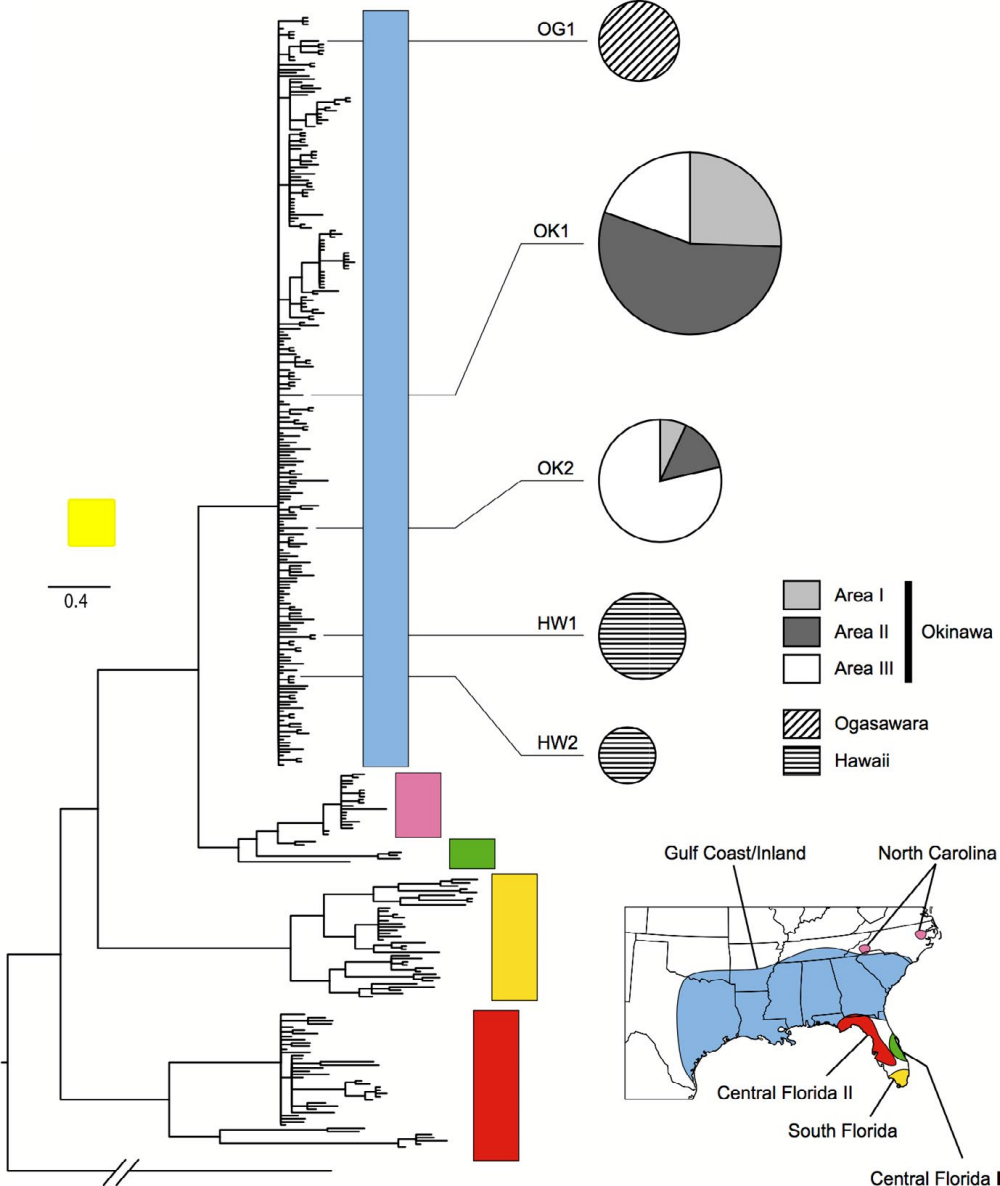
Bayesian ND2 phylogeny using Okinawan, Ogasawaran, and Hawaiian populations in addition to haplotypes used by Campbell‐ Staton et al. (2012) and Hayashi et al. (2009). The major branches with high posterior probabilities of the Bayesian inference method (>0.99) are indicated in bold. The map was redrawn from Campbell‐Staton et al. (2012)

We determined the partial sequences of mitochondrial DNA ND2 (900 bp) from 61 *A. carolinensis* specimens (45 from Okinawa, seven from Ogasawara, and nine from Hawaii) and detected five haplotypes (OK1 and OK2 from Okinawa, OG1 from Ogasawara, and HW1 and HW2 from Hawaii). These haplotypes were deposited in the DDBJ/GenBank (accession number LC033481‐5). The Bayesian phylogeny of ND2 is shown in Figure 2. All haplotypes found in Okinawa, Ogasawara, and Hawaii were included in a Gulf Coast/Inland clade, including the sequences from North Florida, Texas, Louisiana, Mississippi, Arkansas, and South Carolina (Figures 2 and S1). The sequence of one haplotype in Okinawa (OK2, *n *=* *14) corresponded completely to Texas (accession number JX524388) and Louisiana (JQ857844), and the sequence of another haplotype (OK1, *n *=* *31) differed by three nucleotides from Louisiana (JQ857744, JQ857785 and JQ857826) and Texas (JX524384). These two haplotypes were found in the three areas on Okinawa.

Only one haplotype in Ogasawara (OG1) was identical to Ogasawara type A (AB473619), as reported by Hayashi et al. (2009). Three nucleotides differed between the OK2 haplotypes and Ogasawara type B (AB473620), although ≥7 nucleotides differed between the OK1 and Ogasawara haplotypes. The sequence of one haplotype from Hawaii (HW2, *n *=* *3) showed complete correspondence to Texas (accession number JX524389 and JX524390), and the sequence of another haplotype (HW1, *n *=* *6) had three nucleotide substitutions compared with Louisiana (JQ857811) and Arkansas (JQ857836). None of the haplotypes from Okinawa Island corresponded to the Hawaiian haplotypes.

### 3.2 Prediction of suitable areas for *A. carolinensis* on Okinawa Island according to those of native populations

**Figure 3:**
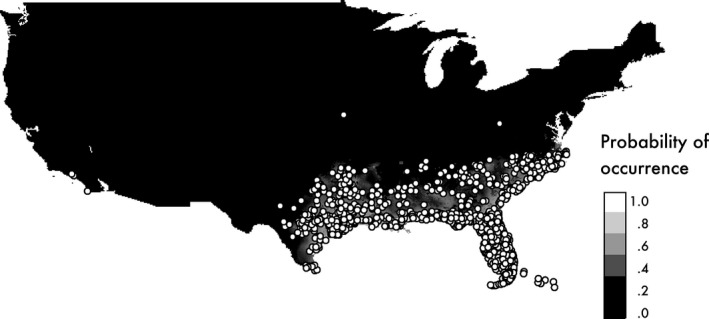
Prediction of suitable areas for *A. carolinensis* by MaxEnt, according to the presence data for North America. Lighter and darker areas indicate areas of high or low suitability, respectively. Points indicate the presence data for *A. carolinensis*

**Figure 4:**
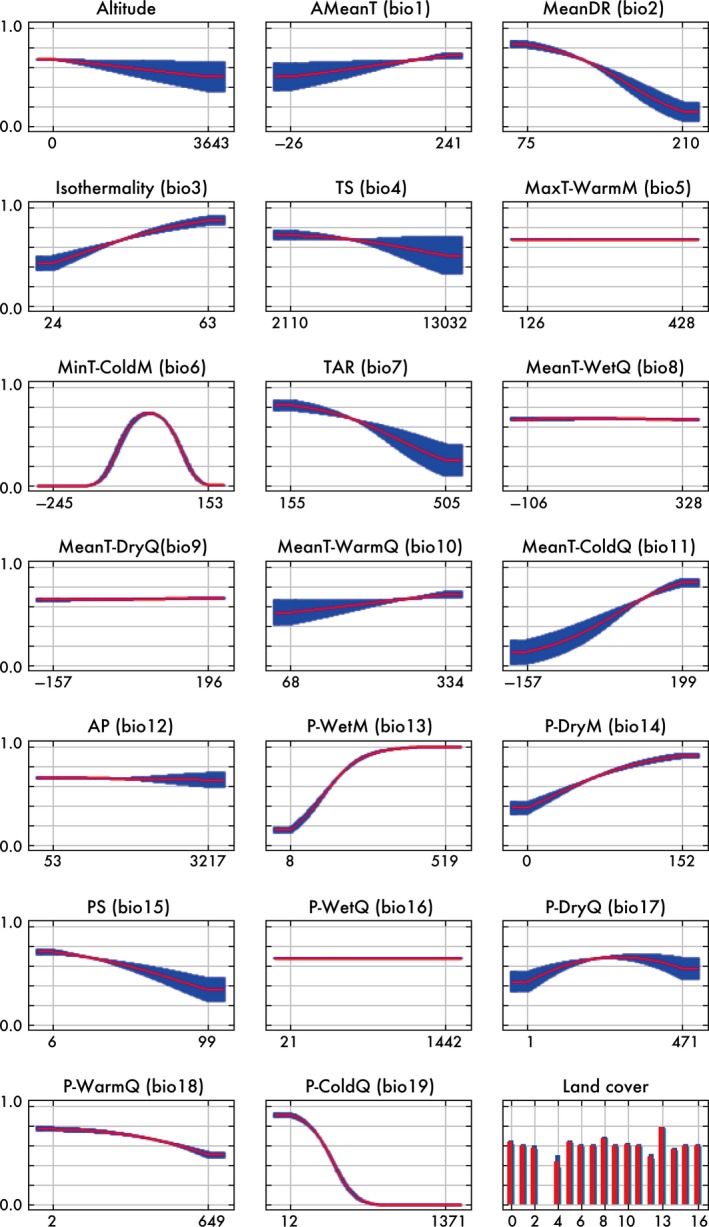
Response curves for the predictions according to the *A. carolinensis* presence data in North America. Units: altitude, m; precipitation, mm; temperature, 0.1°C. The long names of the bioclimatic variables are abbreviated as follows: T, Temperature; P, Precipitation; D, Diurnal; R, Range; S, Seasonality; A, Annual; M, Month; and Q, Quarter. Warm, Cold, Wet, and Dry indicate Warmest, Coldest, Wettest, and Driest, respectively (Table S2). Land‐cover types are used as categorical variables (Table S3). Red and blue represent the mean and one standard deviation, respectively

**Figure 5:**
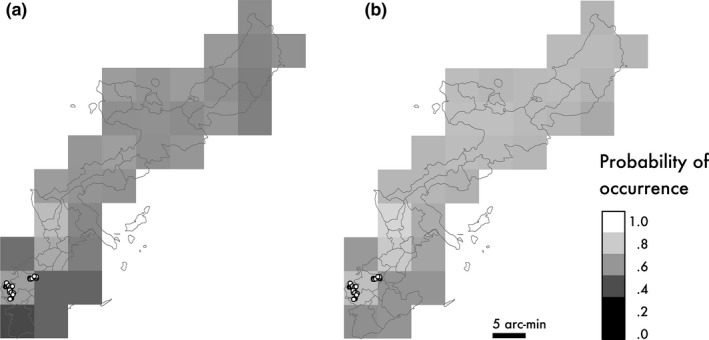
MaxEnt prediction of suitable areas for *A. carolinensis* in Okinawa Island according to the presence data for North America. Lighter and darker areas indicate high or low suitability, respectively. Points indicate the presence distribution of *A. carolinensis*. (a) prediction using all parameters, (b) prediction omitting mean diurnal range and precipitation of warmest quarter

The accuracy of the MaxEnt prediction was very high (mean AUC = 0.931). High‐suitability values included those of southeast North America, which corresponds to their present distributions (Figure 3). Annual mean temperature (bio1) was the most significant contributor to the prediction; followed by land cover (46.5% and 14.4% in Percent contribution in Table 1). Minimum temperature in coldest month (bio6) contributed only 4.8% to the prediction; however, its distribution had a significant effect on the accuracy of prediction (36.1% in permutation importance in Table 1). The jackknife test shows that annual mean temperature (bio1), minimum temperature in coldest month (bio6), and mean temperature in coldest quarter (bio11) were informative variables when we used only one variable to explain the distribution of *A. carolinensis* (Fig. S2). Suitability values increased as annual mean temperature increased and were higher for urban and built‐up areas (label 13) (Figure 4). When suitable areas on Okinawa Island were predicted according to those for North American populations, high suitability values encompassed the entirety of Okinawa Island, although the present distribution of introduced populations was limited to the southern region (Figure 5a).

**Table 1:** Percent contribution and permutation importance of each environmental variable for prediction based on *A. carolinensis* presence data from North America

Variable	Percent contribution	Permutation importance
Annual mean temperature (bio1)	46.5	1.2
Land cover	14.4	2.1
Altitude	11.4	0.3
Precipitation of warmest quarter (bio18)	5.4	0.5
Min temperature of coldest month (bio6)	4.8	36.1
Precipitation of driest quarter (bio17)	2.8	2.1
Temperature annual range (bio7)	2.5	5
Precipitation of driest month (bio14)	2.3	4.7
Precipitation seasonality (bio15)	1.9	1.1
Mean temperature of coldest quarter (bio11)	1.8	9.3
Temperature seasonality (bio4)	1.4	1.1
Mean diurnal range (bio2)	1	4.8
Mean temperature of warmest quarter (bio10)	1	0.2
Precipitation of coldest quarter (bio19)	0.9	15.7
Mean temperature of wettest quarter (bio8)	0.6	0
Precipitation of wettest month (bio13)	0.5	12
Mean temperature of driest quarter (bio9)	0.3	0
Precipitation of wettest quarter (bio16)	0.3	0
Annual precipitation (bio12)	0.2	0
Isothermality (bio3)	0.2	3.7
Max temperature of warmest month (bio5)	0	0

These values are the averages of 100 simulations

In the above prediction, mean diurnal range (bio2) and precipitation of warmest quarter (bio18) were outside the training range in North America, which might decrease the accuracy of the prediction for Okinawa Island. When we excluded these parameters for the prediction, highly suitable areas in North America and the effects of environmental variables were similar, but the suitability in Okinawa Island was higher compared with those in the prediction for all parameters (Figure 5b). The values of Mean Diurnal Range (bio2) in Okinawa Island were lower compared with those of North America, and habitat suitability increased when the value of bio2 was decreased (Figure 4). The values of precipitation of warmest quarter (bio18) in the northern region of Okinawa Island were higher compared with those of North America, and habitat suitability decreased with increasing the value of bio18 (Figure 4).

## 4 Discussion

*A. carolinensis* on Okinawa Island originated from the western region of the Gulf Coast and inland areas of the United States (Figure 2). The MaxEnt predictions based on the native populations of North American show that almost all of the area of Okinawa Island was a suitable habitat for *A. carolinensis* (Figure 5), although the introduced populations in Okinawa did not expand their range from the restricted area. As we did not use a bias file for the MaxEnt prediction based on the native populations of North American, the prediction would reflect the both of habitat suitability and sampling effort biases (Guillera‐Arroita et al., 2015). However, it should not affect the conclusion that Okinawa was suitable for *A. carolinensis* because the use of bias file tends to expand the range of suitable areas. Therefore, *A. carolinensis* may have the potential to expand its distribution throughout Okinawa Island.

### 4.1 Origin of invader populations

The nucleotide sequences of *A. carolinensis* from Okinawa were highly similar to those of anoles from Texas and Louisiana and were substantially different from those found on Ogasawara Islands and Hawaii (Figures 2 and S1). We conclude that a portion of the *A. carolinensis* population introduced into Okinawa Island might not have come directly from the Ogasawara Islands. High‐density areas exist on Okinawa Island near Naha Airport, around harbors, Japan Self‐Defense Force bases, and United States military bases. It was not surprising that *A. carolinensis* was introduced multiple times through these transportation routes. Further, *A. carolinensis* is associated with the pet trade. *A. carolinensis* on Ogasawara Islands was considered to have been introduced by the United States Army from Guam in cargo, released as pets, or both (Lever, 2003; Toda et al., 2009). Unfortunately, samples from Guam were not available, and therefore, this possible route cannot be identified. We used Hawaiian *A. carolinensis*, because there are 11 United States military bases are located in Hawaii, although Okinawan and Hawaiian individuals were genetically different. One or several Okinawan individuals may have been introduced directly from North America.

### 4.2 Factors restricting the expansion of the range of introduced *A. carolinensis* on Okinawa Island

Annual mean temperatures and land cover largely contributed to the MaxEnt prediction (Table 1). Annual mean temperature, minimum temperature of coldest month, and mean temperature in coldest quarter were useful information to explain the distribution of native populations by itself (Fig. S2). The values of precipitation of warmest quarter in the northern region of Okinawa were higher compared with those in North America, which reduced habitat suitability on Okinawa Island. There are no geographic barriers to prevent expansion from the present distribution on Okinawa Island. *A. carolinensis* can move long distance by human transportation (Lever, 2003; Toda et al., 2009) or by predatory birds (Chiba, 2014). We considered that *A. carolinensis* had a restricted distribution in the southern region of Okinawa Island based on a previous survey in which lizards were captured using adhesive traps and observational data were collected from citizens (Naha Nature Conservation Office of the Ministry of the Environment Japan, 2010, 2011, 2012). Reports on sightings and interviews with residents indicated the presence of *A. carolinensis* in the central cities on Okinawa Island, but this information was not reliable. In a recent (2015–2016) island‐wide survey on anole's distribution range, citizens provided eyewitness accounts on several individuals in the southern and central regions of Okinawa island (Kitanakagusuku‐son, Chatan‐cho, Okinawa‐shi, and Nago‐shi); however, surveys using adhesive traps and line census did not record them in these regions (S. Kimura et al., 2016, an unpublished report at the 55th Annual Meeting of the Herpetological Society of Japan). Thus, *A. carolinensis* has the potential to expand its distribution northward but has not yet become established in the central region of Okinawa island. Therefore, we considered possible explanations of why the introduced populations did not expand throughout Okinawa Island as follows: (1) resistance to low temperature, preference for forests, or both might be lower compared with the native populations by a founder effect, (2) high precipitation of warmest quarter in Okinawa Island might be unsuitable, and (3) distributions of predators and competitors on Okinawa Island might restrict expansion.

#### 4.2.1 Lower resistance to low temperatures and reduced preference for closed forests

The MaxEnt predictions suggest that the native *A. carolinensis* populations preferred a high annual mean temperature and urban and built‐up areas (Figure 4). When we used the presence data of Okinawa populations, the MaxEnt prediction suggests that the introduced populations preferred a high annual mean temperature and urban areas (Table S4, and Figs. S3 and S4). *A. carolinensis* potentially adapts to the colder temperatures of northern Okinawa Island (Figure 5), because winters in Texas, which is considered an original habitat of the Okinawan populations (Figure 2), are colder compared with those of Okinawa Island. However, the Okinawan populations may have a lower resistance to low temperatures compared with those of the native populations because of genetic differences derived through founder effects. The mean winter temperature of Okinawa Island is lower compared with those of the invasion areas of *A. carolinensis* (e.g., Hawaii, Guam, and the Ogasawara Islands). The low temperature in the northern region of Okinawa might be unsuitable for the introduced *A. carolinensis*. However, a colder temperature may not restrict the expansion of the introduced *A. carolinensis* in the future, because they may adapt to colder temperatures, or cold‐adapted individuals might be introduced in the future. Further, increasing temperatures caused by global warming increase the probability of expansion.

Land cover (urban and built‐up areas) was found to be suitable for the native *A. carolinensis* populations (Table 1 and Figure 4). In the prediction, the evergreen broad‐leaf forest was also judged suitable in native areas (Figure 4), which resulted in high suitability of the evergreen broad‐leaf forest in the northern region of Okinawa Island (Figure 5). However, the evergreen broad‐leaf forests occupy very small areas in their native locations (the central and northern regions of Florida; Fig. S5). Thus, the founder individuals on Okinawa Island may not have adapted to evergreen broad‐leaf forests.

Similar to its ancestral species *A. porcatus* (Glor et al., 2005) in its original habitat, *A. carolinensis* is found more frequently in open areas outside forests than inside forests in its original habitat (Rodríguez‐Schettino, 2012). On Okinawa Island, *A. carolinensis* is frequently found in urbanized, open areas, or both, such as gardens and roadside trees near airports. For example, the catch per unit effort [(captured individuals)/(traps days)] for *A. carolinensis* on Okinawa Island was higher in open areas compared with those in forested areas (Ishikawa et al., 2012). These open areas provide a sunny and warm environment that allows the anoles to maintain their body temperature, which is particularly critical in winter (Jenssen et al., 1996). The few rural areas and closed forests in the central and northern regions of Okinawa Island might be unsuitable for introduced *A. carolinensis*. The expansion of open areas by human activities such as deforestation could promote the expansion of *A. carolinensis* on Okinawa Island.

#### 4.2.2 Effects of high precipitation in summer and distributions of predators and competitors

Precipitation of warmest quarter in the northern region of Okinawa Island was higher compared with those of the training data of the analysis of North America. Thus, the slope of the true response curve of precipitation of warmest quarter may dramatically decrease at high precipitation values that were not included in the training data of North America, which caused a greater reduction of habitat suitability in the northern region of Okinawa Island. Compared with the established invasion areas of *A. carolinensis*, precipitation data during the summer in Hawaii and the Ogasawara Islands were lower compared with those of the northern region of Okinawa Island, although the precipitation in Guam during summer tends to be higher compared with that of Okinawa Island. Although we were unable to conclude that high precipitation during summer in Okinawa Island simply restricted the expansion of the range of the introduced populations, it might affect the distribution of introduced populations.

We did not use predator and competitor distribution data in our predictions because these data are not available. Toda et al. (2009) suggested that *A. carolinensis* introduced to the Ogasawara Islands could increase their population density, because there are no strong predators and competitors. A variety of predators such as cats, snakes, and birds, and potential competitors such as the green grass lizard *Takydromus smaragdinus*, the Okinawan tree lizard *Japalura polygonata*, the common house gecko *Hemidactylus frenatus*, and the Hokou gecko *Gekko hokouensis* inhabit Okinawa Island (Naha Regional Environmental Office of the Ministry of the Environment 2012; Ishikawa et al., 2012). If their distributions are biased to closed forests in the central and northern regions of Okinawa Island, they may act as factors that can restrict the range of expansion of *A. carolinensis*. The prediction of ecologically suitable areas using predator and competitor data should be performed if these data become available in the future.

## 5 Conclusion

*A. carolinensis* was introduced to Okinawa Island more than 25 years ago, yet its distribution remains restricted to its southern region. Here, we used a combination genetic and distribution‐modeling techniques to determine whether introduced *A. carolinensis* has the potential to expand its distribution on Okinawa Island. This species was likely introduced to Okinawa Island multiple times and originated in the western part of Gulf Coast and inland area, USA. The species distribution model reveals that the entirety of Okinawa Island is suitable for supporting *A. carolinensis*. Therefore, *A. carolinensis* should have the potential to expand its distribution throughout Okinawa Island. Annual mean temperature and land cover had a great effect on habitat suitability. Lower resistance to lower temperatures, reduced preference for closed forests, or both, in introduced populations compared with native populations through a founder effect may restrict the range of expansion of introduced *A. carolinensis*. Further, high precipitation in summer as well as the distributions of predators and competitors may restrict the range expansion of introduced *A. carolinensis*.

## Conflict of Interest

None declared.
