# Supplementary material for: Factors restricting the range expansion of the invasive green anole Anolis carolinensis on Okinawa Island, Japan
Source: Ecol Evol. 2017 May 10;7(12):4357–66. doi: 10.1002/ece3.3002 (PMC5478079; doi:10.1002/ece3.3002)
Supplement: Supplementary file 1 [file ECE3-7-4357-s001.docx]

**Table S1.** Sampling localities and GenBank accession numbers for mtDNA haplotypes used in this study.

| Locality | Accession number | Reference |
| --- | --- | --- |
| Japan: Ogasawara Islands | AB473620 | Hayashi et al. (2009) |
| Japan: Ogasawara Islands | AB473619 | Hayashi et al. (2009) |
| USA: Jacksonville | AY902434 | Glor et al. (2005) |
| USA: Gainesville | AY902433 | Glor et al. (2005) |
| USA: Gainesville | AY902432 | Glor et al. (2005) |
| USA: Inverness | AY902431 | Glor et al. (2005) |
| USA: Inverness | AY902430 | Glor et al. (2005) |
| USA: Okeechobee | AY902429 | Glor et al. (2005) |
| USA: Nassau, FL | JQ857918 | Tollis et al. (2012) |
| USA: Nassau, FL | JQ857917 | Tollis et al. (2012) |
| USA: Augusta, GA | JQ857916 | Tollis et al. (2012) |
| USA: Augusta, GA | JQ857915 | Tollis et al. (2012) |
| USA: Aiken, SC | JQ857914 | Tollis et al. (2012) |
| USA: Nassau, FL | JQ857913 | Tollis et al. (2012) |
| USA: Aiken, SC | JQ857912 | Tollis et al. (2012) |
| USA: Aiken, SC | JQ857911 | Tollis et al. (2012) |
| USA: Nassau, FL | JQ857910 | Tollis et al. (2012) |
| USA: Augusta, GA | JQ857909 | Tollis et al. (2012) |
| USA: Augusta, GA | JQ857908 | Tollis et al. (2012) |
| USA: Augusta, GA | JQ857907 | Tollis et al. (2012) |
| USA: Augusta, GA | JQ857906 | Tollis et al. (2012) |
| USA: Augusta, GA | JQ857905 | Tollis et al. (2012) |
| USA: Augusta, GA | JQ857904 | Tollis et al. (2012) |
| USA: Augusta, GA | JQ857903 | Tollis et al. (2012) |
| USA: Augusta, GA | JQ857902 | Tollis et al. (2012) |
| USA: Aiken, SC | JQ857901 | Tollis et al. (2012) |
| USA: Augusta, GA | JQ857900 | Tollis et al. (2012) |
| USA: Augusta, GA | JQ857899 | Tollis et al. (2012) |
| USA: Augusta, GA | JQ857898 | Tollis et al. (2012) |
| USA: Aiken, SC | JQ857897 | Tollis et al. (2012) |
| USA: Aiken, SC | JQ857896 | Tollis et al. (2012) |
| USA: Aiken, SC | JQ857895 | Tollis et al. (2012) |
| USA: Nassau, FL | JQ857894 | Tollis et al. (2012) |
| USA: Nassau, FL | JQ857893 | Tollis et al. (2012) |
| USA: Everglades City, FL | JQ857892 | Tollis et al. (2012) |
| USA: Everglades City, FL | JQ857891 | Tollis et al. (2012) |
| USA: Everglades City, FL | JQ857890 | Tollis et al. (2012) |
| USA: Everglades City, FL | JQ857888 | Tollis et al. (2012) |
| USA: Everglades City, FL | JQ857887 | Tollis et al. (2012) |
| USA: Everglades City, FL | JQ857886 | Tollis et al. (2012) |
| USA: Everglades City, FL | JQ857885 | Tollis et al. (2012) |
| USA: Everglades City, FL | JQ857884 | Tollis et al. (2012) |
| USA: Everglades City, FL | JQ857883 | Tollis et al. (2012) |
| USA: High Springs, FL | JQ857882 | Tollis et al. (2012) |
| USA: Cooks Hammock, FL | JQ857881 | Tollis et al. (2012) |
| USA: Mayo, FL | JQ857880 | Tollis et al. (2012) |
| USA: Cooks Hammock, FL | JQ857879 | Tollis et al. (2012) |
| USA: Cooks Hammock, FL | JQ857878 | Tollis et al. (2012) |
| USA: High Springs, FL | JQ857877 | Tollis et al. (2012) |
| USA: Cooks Hammock, FL | JQ857876 | Tollis et al. (2012) |
| USA: High Springs, FL | JQ857875 | Tollis et al. (2012) |
| USA: Cooks Hammock, FL | JQ857873 | Tollis et al. (2012) |
| USA: Hines, FL | JQ857872 | Tollis et al. (2012) |
| USA: Hines, FL | JQ857871 | Tollis et al. (2012) |
| USA: Cooks Hammock, FL | JQ857870 | Tollis et al. (2012) |
| USA: Holden Beach, NC | JQ857869 | Tollis et al. (2012) |
| USA: Holden Beach, NC | JQ857868 | Tollis et al. (2012) |
| USA: Bogue, NC | JQ857867 | Tollis et al. (2012) |
| USA: Bogue, NC | JQ857866 | Tollis et al. (2012) |
| USA: Morehead City, NC | JQ857865 | Tollis et al. (2012) |
| USA: Morehead City, NC | JQ857864 | Tollis et al. (2012) |
| USA: Cooks Hammock, FL | JQ857863 | Tollis et al. (2012) |
| USA: Mayo, FL | JQ857862 | Tollis et al. (2012) |
| USA: Georgetown, SC | JQ857861 | Tollis et al. (2012) |
| USA: Jacksonville, GA | JQ857860 | Tollis et al. (2012) |
| USA: North Santee, SC | JQ857859 | Tollis et al. (2012) |
| USA: Bibb County, AL | JQ857858 | Tollis et al. (2012) |
| USA: North Santee, SC | JQ857857 | Tollis et al. (2012) |
| USA: Blount County, TN | JQ857856 | Tollis et al. (2012) |
| USA: Darien, GA | JQ857855 | Tollis et al. (2012) |
| USA: Wood County, TX | JQ857854 | Tollis et al. (2012) |
| USA: Thibodaux, LA | JQ857853 | Tollis et al. (2012) |
| USA: Blount County, TN | JQ857852 | Tollis et al. (2012) |
| USA: Holden Beach, NC | JQ857851 | Tollis et al. (2012) |
| USA: Sorrento, LA | JQ857850 | Tollis et al. (2012) |
| USA: Lugoff, SC | JQ857849 | Tollis et al. (2012) |
| USA: Mobile, AL | JQ857848 | Tollis et al. (2012) |
| USA: Bibb County, AL | JQ857847 | Tollis et al. (2012) |
| USA: Johnson County, GA | JQ857846 | Tollis et al. (2012) |
| USA: North Santee, SC | JQ857845 | Tollis et al. (2012) |
| USA: Thibodaux, LA | JQ857844 | Tollis et al. (2012) |
| USA: Bogue, NC | JQ857843 | Tollis et al. (2012) |
| USA: Jacksonville, GA | JQ857842 | Tollis et al. (2012) |
| USA: Blount County, TN | JQ857841 | Tollis et al. (2012) |
| USA: Thibodaux, LA | JQ857840 | Tollis et al. (2012) |
| USA: Wood County, TX | JQ857839 | Tollis et al. (2012) |
| USA: Blount County, TN | JQ857838 | Tollis et al. (2012) |
| USA: Blount County, TN | JQ857837 | Tollis et al. (2012) |
| USA: Ogden, AR | JQ857836 | Tollis et al. (2012) |
| USA: Thibodaux, LA | JQ857835 | Tollis et al. (2012) |
| USA: Sorrento, LA | JQ857834 | Tollis et al. (2012) |
| USA: Lugoff, SC | JQ857833 | Tollis et al. (2012) |
| USA: Ogden, AR | JQ857832 | Tollis et al. (2012) |
| USA: Thibodaux, LA | JQ857831 | Tollis et al. (2012) |
| USA: Bibb County, AL | JQ857829 | Tollis et al. (2012) |
| USA: Blount County, TN | JQ857828 | Tollis et al. (2012) |
| USA: Springfield, LA | JQ857827 | Tollis et al. (2012) |
| USA: Jay, LA | JQ857826 | Tollis et al. (2012) |
| USA: Crenshaw County, AL | JQ857825 | Tollis et al. (2012) |
| USA: Blond, LA | JQ857824 | Tollis et al. (2012) |
| USA: Bogue, NC | JQ857823 | Tollis et al. (2012) |
| USA: Thibodaux, LA | JQ857822 | Tollis et al. (2012) |
| USA: Darien, GA | JQ857821 | Tollis et al. (2012) |
| USA: Blount County, TN | JQ857820 | Tollis et al. (2012) |
| USA: Springfield, LA | JQ857819 | Tollis et al. (2012) |
| USA: Mobile, AL | JQ857818 | Tollis et al. (2012) |
| USA: French Settlement, LA | JQ857817 | Tollis et al. (2012) |
| USA: Wood County, TX | JQ857816 | Tollis et al. (2012) |
| USA: Jacksonville, GA | JQ857815 | Tollis et al. (2012) |
| USA: Blount County, TN | JQ857814 | Tollis et al. (2012) |
| USA: High Springs, FL | JQ857813 | Tollis et al. (2012) |
| USA: Thibodaux, LA | JQ857812 | Tollis et al. (2012) |
| USA: Thibodaux, LA | JQ857811 | Tollis et al. (2012) |
| USA: Lugoff, SC | JQ857810 | Tollis et al. (2012) |
| USA: Blond, LA | JQ857809 | Tollis et al. (2012) |
| USA: Jay, LA | JQ857808 | Tollis et al. (2012) |
| USA: Blond, LA | JQ857807 | Tollis et al. (2012) |
| USA: Springfield, LA | JQ857806 | Tollis et al. (2012) |
| USA: Bibb County, AL | JQ857805 | Tollis et al. (2012) |
| USA: Blount County, TN | JQ857804 | Tollis et al. (2012) |
| USA: Blond, LA | JQ857803 | Tollis et al. (2012) |
| USA: Killian, LA | JQ857802 | Tollis et al. (2012) |
| USA: Blount County, TN | JQ857801 | Tollis et al. (2012) |
| USA: Blond, LA | JQ857800 | Tollis et al. (2012) |
| USA: Morehead City, NC | JQ857799 | Tollis et al. (2012) |
| USA: Jay, LA | JQ857798 | Tollis et al. (2012) |
| USA: Wood County, TX | JQ857797 | Tollis et al. (2012) |
| USA: Darien, GA | JQ857796 | Tollis et al. (2012) |
| USA: Lugoff, SC | JQ857795 | Tollis et al. (2012) |
| USA: Mobile, AL | JQ857794 | Tollis et al. (2012) |
| USA: Jay, LA | JQ857793 | Tollis et al. (2012) |
| USA: Springfield, LA | JQ857792 | Tollis et al. (2012) |
| USA: Springfield, LA | JQ857790 | Tollis et al. (2012) |
| USA: Blount County, TN | JQ857789 | Tollis et al. (2012) |
| USA: Holden Beach, NC | JQ857788 | Tollis et al. (2012) |
| USA: Thibodaux, LA | JQ857787 | Tollis et al. (2012) |
| USA: Holden Beach, NC | JQ857786 | Tollis et al. (2012) |
| USA: Killian, LA | JQ857785 | Tollis et al. (2012) |
| USA: Blount County, TN | JQ857784 | Tollis et al. (2012) |
| USA: Dodge County, GA | JQ857783 | Tollis et al. (2012) |
| USA: Blount County, TN | JQ857782 | Tollis et al. (2012) |
| USA: Morehead City, NC | JQ857781 | Tollis et al. (2012) |
| USA: North Santee, SC | JQ857780 | Tollis et al. (2012) |
| USA: Wood County, TX | JQ857779 | Tollis et al. (2012) |
| USA: Blount County, TN | JQ857778 | Tollis et al. (2012) |
| USA: Bibb County, AL | JQ857777 | Tollis et al. (2012) |
| USA: Lugoff, SC | JQ857776 | Tollis et al. (2012) |
| USA: Mobile, AL | JQ857775 | Tollis et al. (2012) |
| USA: High Springs, FL | JQ857774 | Tollis et al. (2012) |
| USA: Wood County, TX | JQ857773 | Tollis et al. (2012) |
| USA: Darien, GA | JQ857772 | Tollis et al. (2012) |
| USA: Baldwin County, AL | JQ857771 | Tollis et al. (2012) |
| USA: Blount County, TN | JQ857770 | Tollis et al. (2012) |
| USA: Bogue, NC | JQ857769 | Tollis et al. (2012) |
| USA: Columbia County, GA | JQ857768 | Tollis et al. (2012) |
| USA: Thibodaux, LA | JQ857767 | Tollis et al. (2012) |
| USA: Blount County, TN | JQ857766 | Tollis et al. (2012) |
| USA: French Settlement, LA | JQ857765 | Tollis et al. (2012) |
| USA: Holden Beach, NC | JQ857764 | Tollis et al. (2012) |
| USA: Jacksonville, GA | JQ857763 | Tollis et al. (2012) |
| USA: Blount County, TN | JQ857762 | Tollis et al. (2012) |
| USA: North Santee, SC | JQ857761 | Tollis et al. (2012) |
| USA: Blount County, TN | JQ857760 | Tollis et al. (2012) |
| USA: Morehead City, NC | JQ857759 | Tollis et al. (2012) |
| USA: Thibodaux, LA | JQ857758 | Tollis et al. (2012) |
| USA: Mobile, AL | JQ857757 | Tollis et al. (2012) |
| USA: Bibb County, AL | JQ857756 | Tollis et al. (2012) |
| USA: Thibodaux, LA | JQ857755 | Tollis et al. (2012) |
| USA: Lugoff, SC | JQ857754 | Tollis et al. (2012) |
| USA: Wood County, TX | JQ857753 | Tollis et al. (2012) |
| USA: Jay, LA | JQ857751 | Tollis et al. (2012) |
| USA: Blount County, TN | JQ857750 | Tollis et al. (2012) |
| USA: Blount County, TN | JQ857749 | Tollis et al. (2012) |
| USA: Wood County, TX | JQ857748 | Tollis et al. (2012) |
| USA: Columbia County, GA | JQ857747 | Tollis et al. (2012) |
| USA: Okeechobee, FL | JQ857746 | Tollis et al. (2012) |
| USA: Sorrento, LA | JQ857745 | Tollis et al. (2012) |
| USA: Thibodaux, LA | JQ857744 | Tollis et al. (2012) |
| USA: Blount County, TN | JQ857743 | Tollis et al. (2012) |
| USA: Lugoff, SC | JQ857742 | Tollis et al. (2012) |
| USA: Blount County, TN | JQ857741 | Tollis et al. (2012) |
| USA: Blount County, TN | JQ857740 | Tollis et al. (2012) |
| USA: Perry County, AL | JQ857739 | Tollis et al. (2012) |
| USA: Blount County, TN | JQ857738 | Tollis et al. (2012) |
| USA: Wilcox County, AL | JQ857737 | Tollis et al. (2012) |
| USA: Baldwin County, AL | JQ857736 | Tollis et al. (2012) |
| USA: Jay, LA | JQ857735 | Tollis et al. (2012) |
| USA: Live Oak, FL | JQ857734 | Tollis et al. (2012) |
| USA: Baldwin County, AL | JQ857733 | Tollis et al. (2012) |
| USA: Blount County, TN | JQ857731 | Tollis et al. (2012) |
| USA: Lugoff, SC | JQ857730 | Tollis et al. (2012) |
| USA: North Santee, SC | JQ857729 | Tollis et al. (2012) |
| USA: Florida, Coral Gables | EU106342 | Kolbe et al. (2007) |
| USA: Florida, Coral Gables | EU106341 | Kolbe et al. (2007) |
| USA: Florida, Coral Gables | EU106340 | Kolbe et al. (2007) |
| USA: Florida, Coral Gables | EU106339 | Kolbe et al. (2007) |
| USA: Florida, Coral Gables | EU106338 | Kolbe et al. (2007) |
| USA: Florida, Coral Gables | EU106337 | Kolbe et al. (2007) |
| USA: Florida, Coral Gables | EU106336 | Kolbe et al. (2007) |
| USA: Florida, Coral Gables | EU106335 | Kolbe et al. (2007) |
| USA: Florida, Coral Gables | EU106334 | Kolbe et al. (2007) |
| USA: Florida, Coral Gables | EU106333 | Kolbe et al. (2007) |
| USA: Florida, Coral Gables | EU106332 | Kolbe et al. (2007) |
| USA: Florida, Coral Gables | EU106330 | Kolbe et al. (2007) |
| USA: Florida, Palatka | EU106329 | Kolbe et al. (2007) |
| USA: Florida, Palatka | EU106328 | Kolbe et al. (2007) |
| USA: Florida, Gainesville | EU106327 | Kolbe et al. (2007) |
| USA: Florida, Bronson | EU106326 | Kolbe et al. (2007) |
| USA: Florida, Mayo | EU106325 | Kolbe et al. (2007) |
| USA: Florida, Chiefland | EU106324 | Kolbe et al. (2007) |
| USA: Florida, Inverness | EU106323 | Kolbe et al. (2007) |
| USA: Hillsborough, FL | KP174776 | Krysko et al. (2015) |
| USA: Hillsborough, FL | KP174775 | Krysko et al. (2015) |
| USA: Hillsborough, FL | KP174774 | Krysko et al. (2015) |
| USA: Hillsborough, FL | KP174773 | Krysko et al. (2015) |
| USA: Hillsborough, FL | KP174772 | Krysko et al. (2015) |
| USA: Mississippi, Wade | JX524427 | Campbell-Staton et al. (2012) |
| USA: Mississippi, Wade | JX524426 | Campbell-Staton et al. (2012) |
| USA: Mississippi, Wade | JX524424 | Campbell-Staton et al. (2012) |
| USA: Florida, Panama City | JX524422 | Campbell-Staton et al. (2012) |
| USA: Florida, Panama City | JX524421 | Campbell-Staton et al. (2012) |
| USA: Florida, Panama City | JX524420 | Campbell-Staton et al. (2012) |
| USA: Florida, Panama City | JX524419 | Campbell-Staton et al. (2012) |
| USA: Florida, Panama City | JX524418 | Campbell-Staton et al. (2012) |
| USA: Florida, Panama City | JX524417 | Campbell-Staton et al. (2012) |
| USA: Florida, Naples | JX524416 | Campbell-Staton et al. (2012) |
| USA: Florida, Naples | JX524415 | Campbell-Staton et al. (2012) |
| USA: Florida, Naples | JX524414 | Campbell-Staton et al. (2012) |
| USA: Florida, Naples | JX524413 | Campbell-Staton et al. (2012) |
| USA: Florida, Palm Beach | JX524408 | Campbell-Staton et al. (2012) |
| USA: North Carolina, Washington | JX524407 | Campbell-Staton et al. (2012) |
| USA: North Carolina, Washington | JX524405 | Campbell-Staton et al. (2012) |
| USA: North Carolina, Washington | JX524404 | Campbell-Staton et al. (2012) |
| USA: North Carolina, Washington | JX524403 | Campbell-Staton et al. (2012) |
| USA: South Carolina, Myrtle Beach | JX524402 | Campbell-Staton et al. (2012) |
| USA: South Carolina, Myrtle Beach | JX524401 | Campbell-Staton et al. (2012) |
| USA: South Carolina, Myrtle Beach | JX524400 | Campbell-Staton et al. (2012) |
| USA: South Carolina, Myrtle Beach | JX524398 | Campbell-Staton et al. (2012) |
| USA: Alabama, Opelika | JX524397 | Campbell-Staton et al. (2012) |
| USA: Alabama, Opelika | JX524396 | Campbell-Staton et al. (2012) |
| USA: Alabama, Opelika | JX524395 | Campbell-Staton et al. (2012) |
| USA: Alabama, Opelika | JX524394 | Campbell-Staton et al. (2012) |
| USA: Alabama, Opelika | JX524393 | Campbell-Staton et al. (2012) |
| USA: Texas, Orange | JX524392 | Campbell-Staton et al. (2012) |
| USA: Texas, Orange | JX524391 | Campbell-Staton et al. (2012) |
| USA: Texas, Orange | JX524390 | Campbell-Staton et al. (2012) |
| USA: Texas, Orange | JX524389 | Campbell-Staton et al. (2012) |
| USA: Texas, Orange | JX524388 | Campbell-Staton et al. (2012) |
| USA: Texas, Brownsville | JX524387 | Campbell-Staton et al. (2012) |
| USA: Texas, Brownsville | JX524386 | Campbell-Staton et al. (2012) |
| USA: Texas, Brownsville | JX524384 | Campbell-Staton et al. (2012) |
| USA: Texas, Brownsville | JX524383 | Campbell-Staton et al. (2012) |
| USA: Tennessee, Pickwick | JX524382 | Campbell-Staton et al. (2012) |
| USA: Tennessee, Pickwick | JX524381 | Campbell-Staton et al. (2012) |
| USA: Tennessee, Pickwick | JX524380 | Campbell-Staton et al. (2012) |
| USA: Tennessee, Pickwick | JX524379 | Campbell-Staton et al. (2012) |
| USA: Tennessee, Pickwick | JX524378 | Campbell-Staton et al. (2012) |
| USA: Floria, Chuluota | JX524377 | Campbell-Staton et al. (2012) |
| USA: Floria, Chuluota | JX524376 | Campbell-Staton et al. (2012) |
| USA: Floria, Chuluota | JX524375 | Campbell-Staton et al. (2012) |
| USA: Floria, Chuluota | JX524374 | Campbell-Staton et al. (2012) |
| USA: Floria, Chuluota | JX524373 | Campbell-Staton et al. (2012) |
| USA: Florida, Jacksonville | JX524372 | Campbell-Staton et al. (2012) |
| USA: Florida, Jacksonville | JX524371 | Campbell-Staton et al. (2012) |
| USA: Florida, Jacksonville | JX524370 | Campbell-Staton et al. (2012) |
| USA: Florida, Jacksonville | JX524369 | Campbell-Staton et al. (2012) |
| USA: Florida, Jacksonville | JX524368 | Campbell-Staton et al. (2012) |
| USA: Georgia, Augusta | JX524367 | Campbell-Staton et al. (2012) |
| USA: Georgia, Augusta | JX524366 | Campbell-Staton et al. (2012) |
| USA: Georgia, Augusta | JX524365 | Campbell-Staton et al. (2012) |
| USA: Georgia, Augusta | JX524364 | Campbell-Staton et al. (2012) |
| USA: Georgia, Augusta | JX524363 | Campbell-Staton et al. (2012) |
| USA: Tennessee, Tallasee | JX524362 | Campbell-Staton et al. (2012) |
| USA: Tennessee, Tallasee | JX524361 | Campbell-Staton et al. (2012) |
| USA: Tennessee, Tallasee | JX524360 | Campbell-Staton et al. (2012) |
| USA: Tennessee, Tallasee | JX524358 | Campbell-Staton et al. (2012) |
| USA: Arkansas, Akadelphia | JX524357 | Campbell-Staton et al. (2012) |
| USA: Arkansas, Akadelphia | JX524356 | Campbell-Staton et al. (2012) |
| USA: Arkansas, Akadelphia | JX524355 | Campbell-Staton et al. (2012) |
| USA: Arkansas, Akadelphia | JX524354 | Campbell-Staton et al. (2012) |
| USA: Arkansas, Akadelphia | JX524353 | Campbell-Staton et al. (2012) |
| USA: Texas, Corpus Christie | JX524351 | Campbell-Staton et al. (2012) |
| USA: Texas, Corpus Christie | JX524350 | Campbell-Staton et al. (2012) |
| USA: Texas, Corpus Christie | JX524348 | Campbell-Staton et al. (2012) |
| USA: Texas, Tyler | JX524347 | Campbell-Staton et al. (2012) |
| USA: Texas, Tyler | JX524346 | Campbell-Staton et al. (2012) |
| USA: Texas, Tyler | JX524345 | Campbell-Staton et al. (2012) |
| USA: Texas, Tyler | JX524344 | Campbell-Staton et al. (2012) |
| USA: Texas, Tyler | JX524343 | Campbell-Staton et al. (2012) |
| USA: Louisiana, Monroe | JX524342 | Campbell-Staton et al. (2012) |
| USA: Louisiana, Monroe | JX524339 | Campbell-Staton et al. (2012) |
| USA: Louisiana, Monroe | JX524338 | Campbell-Staton et al. (2012) |
| USA: Louisiana, Lafayette | JX524337 | Campbell-Staton et al. (2012) |
| USA: Louisiana, Lafayette | JX524335 | Campbell-Staton et al. (2012) |
| USA: Louisiana, Lafayette | JX524334 | Campbell-Staton et al. (2012) |
| USA: Louisiana, Lafayette | JX524333 | Campbell-Staton et al. (2012) |
| USA: Florida, ChipolaRiver | JX524332 | Campbell-Staton et al. (2012) |
| USA: Florida, ChipolaRiver | JX524331 | Campbell-Staton et al. (2012) |
| USA: Florida, ChipolaRiver | JX524330 | Campbell-Staton et al. (2012) |
| USA: Florida, ChipolaRiver | JX524329 | Campbell-Staton et al. (2012) |
| USA: Florida, ChipolaRiver | JX524328 | Campbell-Staton et al. (2012) |
| USA: Florida, ChipolaRiver | JX524327 | Campbell-Staton et al. (2012) |
| USA: Florida, ChipolaRiver | JX524326 | Campbell-Staton et al. (2012) |
| USA: Florida, CashBayou | JX524325 | Campbell-Staton et al. (2012) |
| USA: Florida, CashBayou | JX524324 | Campbell-Staton et al. (2012) |
| USA: Florida, LottsMillCreek | JX524323 | Campbell-Staton et al. (2012) |
| USA: Florida, ChipolaCutoff | JX524322 | Campbell-Staton et al. (2012) |
| USA: Florida, Blountstown | JX524321 | Campbell-Staton et al. (2012) |
| USA: Florida, Scanlon | JX524320 | Campbell-Staton et al. (2012) |
| USA: Florida, Econfina | JX524319 | Campbell-Staton et al. (2012) |
| USA: Florida, Econfina | JX524318 | Campbell-Staton et al. (2012) |
| USA: Florida, Econfina | JX524317 | Campbell-Staton et al. (2012) |
| USA: Florida, Econfina | JX524316 | Campbell-Staton et al. (2012) |
| USA: Florida, LebanonStation | JX524315 | Campbell-Staton et al. (2012) |
| USA: Florida, FloralCity | JX524313 | Campbell-Staton et al. (2012) |
| USA: Florida, FloralCity | JX524312 | Campbell-Staton et al. (2012) |
| USA: Florida | JX524310 | Campbell-Staton et al. (2012) |
| USA: Texas, DeerPark, Houston | JX524308 | Campbell-Staton et al. (2012) |
| USA: Texas, DeerPark, Houston | JX524307 | Campbell-Staton et al. (2012) |
| USA: Texas, DeerPark, Houston | JX524306 | Campbell-Staton et al. (2012) |
| USA: Texas, DeerPark, Houston | JX524305 | Campbell-Staton et al. (2012) |
| USA: Florida, RedRoad | JX524304 | Campbell-Staton et al. (2012) |
| USA: Florida, RedRoad | JX524303 | Campbell-Staton et al. (2012) |
| USA: Florida, RedRoad | JX524302 | Campbell-Staton et al. (2012) |
| USA: Florida, RedRoad | JX524301 | Campbell-Staton et al. (2012) |
| USA: Florida, RedRoad | JX524300 | Campbell-Staton et al. (2012) |
| USA: Florida, UMiami | JX524299 | Campbell-Staton et al. (2012) |
| USA: Florida, Parkland | JX524298 | Campbell-Staton et al. (2012) |
| USA: Florida, Parkland | JX524297 | Campbell-Staton et al. (2012) |
| USA: Florida, Parkland | JX524296 | Campbell-Staton et al. (2012) |
| USA: Florida, HighlandsHammocks | JX524295 | Campbell-Staton et al. (2012) |
| USA: Florida, HighlandsHammocks | JX524294 | Campbell-Staton et al. (2012) |
| USA: Florida, HighlandsHammocks | JX524293 | Campbell-Staton et al. (2012) |
| USA: Florida, HighlandsHammocks | JX524292 | Campbell-Staton et al. (2012) |
| USA: Florida, HighlandsHammocks | JX524291 | Campbell-Staton et al. (2012) |
| USA: Georgia, Augusta | JX524290 | Campbell-Staton et al. (2012) |
| USA: Florida, RedRoad | JX524289 | Campbell-Staton et al. (2012) |

**Table S2.** Code values of bioclimatic data in WorldClim.

| Value (bio-) | Label |
| --- | --- |
| 1 | Annual Mean Temperature |
| 2 | Mean Diurnal Range (Mean of monthly (max temp – min temp)) |
| 3 | Isothermality ((bio2/bio7)*100) |
| 4 | Temperature Seasonality (standard deviation*100) |
| 5 | Max Temperature of Warmest Month |
| 6 | Min Temperature of Coldest Month |
| 7 | Temperature Annual Range (bio5 – bio6) |
| 8 | Mean Temperature of Wettest Quarter |
| 9 | Mean Temperature of Driest Quarter |
| 10 | Mean Temperature of Warmest Quarter |
| 11 | Mean Temperature of Coldest Quarter |
| 12 | Annual Precipitation |
| 13 | Precipitation of Wettest Month |
| 14 | Precipitation of Driest Month |
| 15 | Precipitation Seasonality (Coefficient of Variation) |
| 16 | Precipitation of Wettest Quarter |
| 17 | Precipitation of Driest Quarter |
| 18 | Precipitation of Warmest Quarter |
| 19 | Precipitation of Coldest Quarter |

**Table S3.** Code values of MODIS land cover data.

| Value | Label |
| --- | --- |
| 0 | Water |
| 1 | Evergreen needle-leaf forest |
| 2 | Evergreen broad-leaf forest |
| 3 | Deciduous needle-leaf forest |
| 4 | Deciduous broad-leaf forest |
| 5 | Mixed forest |
| 6 | Closed shrub-lands |
| 7 | Open shrub-lands |
| 8 | Woody savannas |
| 9 | Savannas |
| 10 | Grassland |
| 11 | Permanent wetlands |
| 12 | Crops-lands |
| 13 | Urban and built-up |
| 14 | Cropland/Natural vegetation mosaic |
| 15 | Snow and ice |
| 16 | Barren or sparsely vegetated |

**Figure S1.** Fine scale of Bayesian ND2 phylogeny. Abbreviation of collecting location are given followed by the accession number (Japan: OG = Ogasawara; USA: AL = Alabama, AR = Arkansas, FL = Florida, GA = Georgia, LA = Louisiana, MS = Mississippi, NC = North Carolina, SC = South Carolina, TN = Tennessee, TX = Texas). Numerals near the branches are Bayesian posterior probabilities (shown when >0.99).


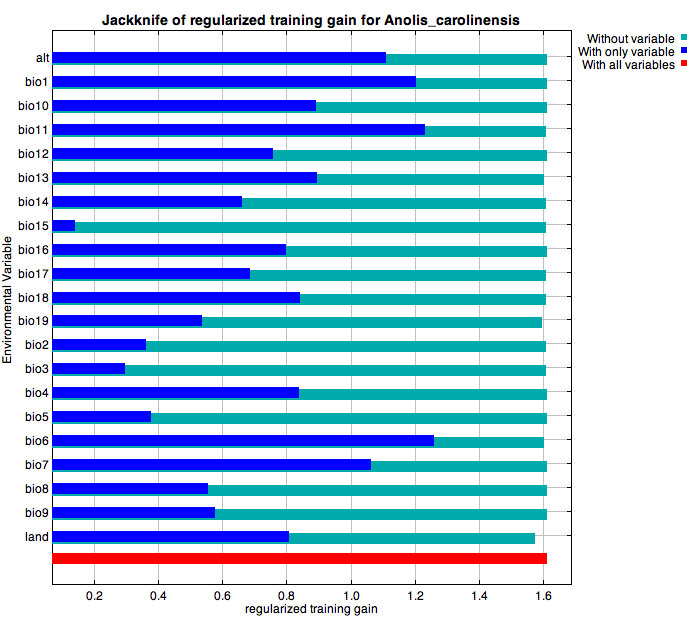


**Figure S2.** The jackknife test shows how an environmental variable affects prediction. The red bar indicates regularized training gain using all environmental variables. The blue bar indicates regularized training gain using only a target variable. The green bar indicates regularized training gain using all environmental variables except for a target variable. alt=altitude, bio1=Annual Mean Temperature, bio2=Mean Diurnal Range, bio3 = Isothermality, bio4 =Temperature Seasonality, bio5=Max Temperature of Warmest Month, bio6=Min Temperature of Coldest Month, bio7=Temperature Annual Range, bio8=Mean Temperature of Wettest Quarter, bio9=Mean Temperature of Driest Quarter, bio10=Mean Temperature of Warmest Quarter, bio11=Mean Temperature of Coldest Quarter, bio12=Annual Precipitation, bio13=Precipitation of Wettest Month, bio14=Precipitation of Driest Month, bio15=Precipitation Seasonality, bio16=Precipitation of Wettest Quarter, bio17=Precipitation of Driest Quarter, bio18=Precipitation of Warmest Quarter, bio19=Precipitation of Coldest Quarter, land=land cover (categorical variable).

***Prediction of ecologically suitable habitats on Okinawa Island according to the Okinawa populations***

If introduced *A. carolinensis* already expanded its distribution to suitable areas on Okinawa Island, the MaxEnt prediction based on the presence data for Okinawa Island will reveal the factors that restrict expansion. Therefore, we used MaxEnt to estimate suitable areas on Okinawa Island according to the presence data of Okinawan populations.

Capture investigation data from high-density areas acquired by the Ministry of the Environment were used ss the presence data for Okinawan populations. Spatial filtering processing was performed by selecting one record within a cell of 0.001 degree to reduce the effects of sampling bias (Kramer-Schadt *et al*. 2013). This spatial filtering reduced the presence data for the high-density area to 36 points, because *A. carolinensis* lives within a very small area on Okinawa Island.

We used climate, altitude, land use, and vegetation data to predict suitable habitats on Okinawa based on the presence data of the Okinawan population. Climate, altitude, and land-use data were obtained from the National Land Numerical Information Service (<http://nlftp.mlit.go.jp/ksj-e/index.html>), and the vegetation map was obtained from the Biodiversity Center of Japan (<http://www.biodic.go.jp/trialSystem/EN/vg/vg.html>). Because most of the 83 climate variables strongly correlated, we selected annual mean temperature, annual precipitation, and global solar irradiance. Of the nine altitude variables, the mean altitude was used. Land-use data comprised 12 land-use types. The vegetation map comprised 902 vegetation types throughout the entirety of Japan and 49 vegetation types in Okinawa Prefecture. These data have different resolutions as follows: average weather 1 km; altitude data 250 m; land-use data 100 m; and vegetation data are shown as polygons. The cell sizes for these data were changed to 0.001 degree using ArcGIS ver. 10.0. The maximum area within a cell was defined as the cell value.

The predicted suitable areas show good correspondence to the present distribution of *A. carolinensis* (Fig. S3). The annual mean temperature and urban areas largely contributed to the predictions (Table S4), and suitability increased as the annual mean temperature increased (Fig. S4). Suitability was higher in urban and artificial land areas (Fig. S4). These results are consistent with those of the prediction using the presence data of for native populations in North America, although this prediction is somewhat problematic. For example, if introduced populations did not reach suitable areas in Okinawa Island for unknown reasons, the prediction according to the presence data for Okinawa Island would lead to a misleading result. Further, the prediction did not account for sampling bias. We acquired data from eyewitness accounts of citizens indicating that introduced *A. carolinensis* was not observed in the northern region of Okinawa Island (Naha Nature Conservation Office of the Ministry of the Environment Japan 2010, 2011, 2012); however, sampling bias may affect the prediction.

**Table S4.** Percent contribution and permutation importance of each environmental variable for prediction based on *A. carolinensis* presence data on Okinawa Island.

| Variable | Percent contribution | Permutation importance |
| --- | --- | --- |
| Annual mean temperature | 56.9 | 94 |
| Vegetation type | 28.3 | 2.6 |
| Land-use type | 9.9 | 1.5 |
| Global solar irradiance | 4 | 0.3 |
| Altitude | 0.7 | 1.5 |
| Annual precipitation | 0.2 | 0.2 |


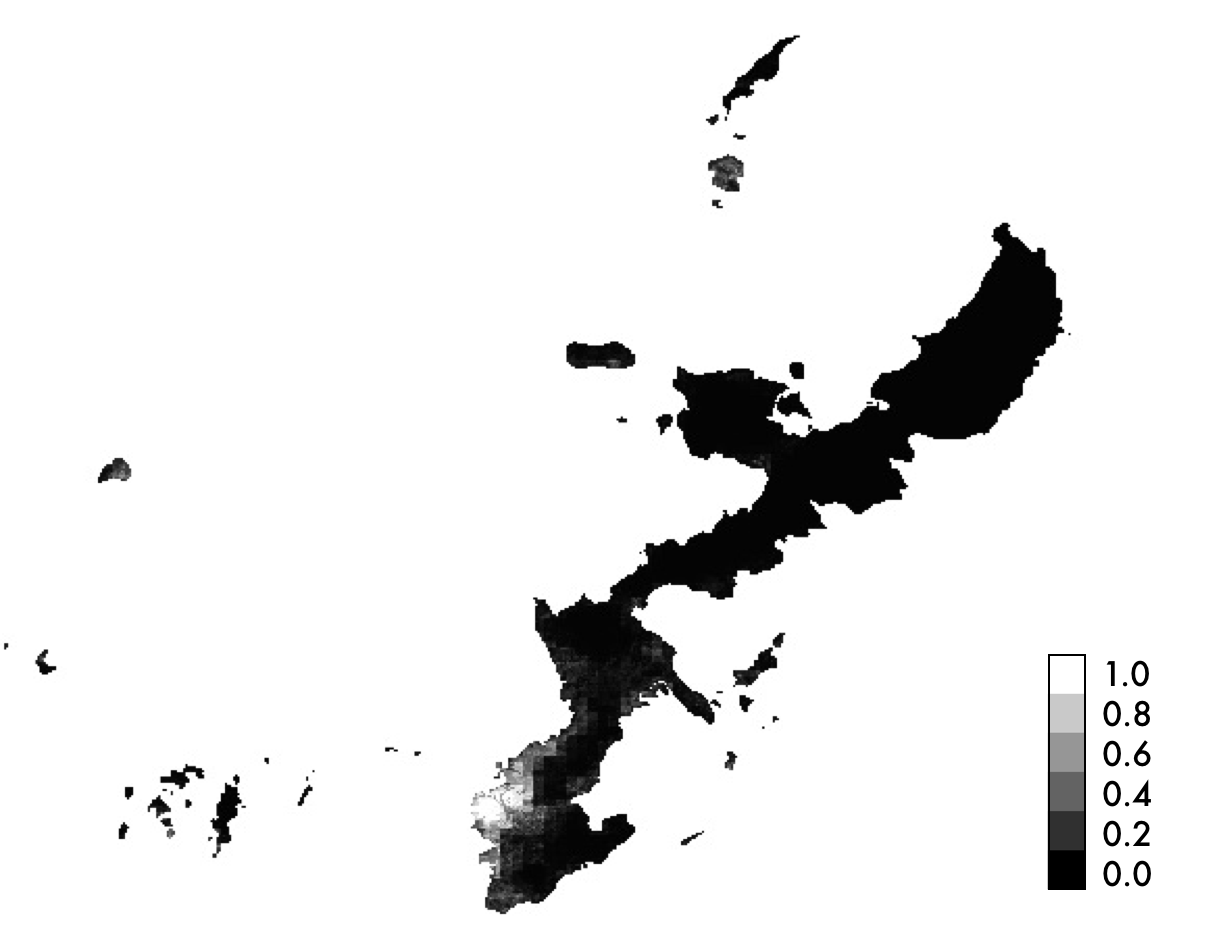


**Figure S3.** MaxEnt prediction of suitable habitats for *A. carolinensis* based on the presence data of Okinawan populations. Lighter and darker areas indicate areas of high and low suitability, respectively.


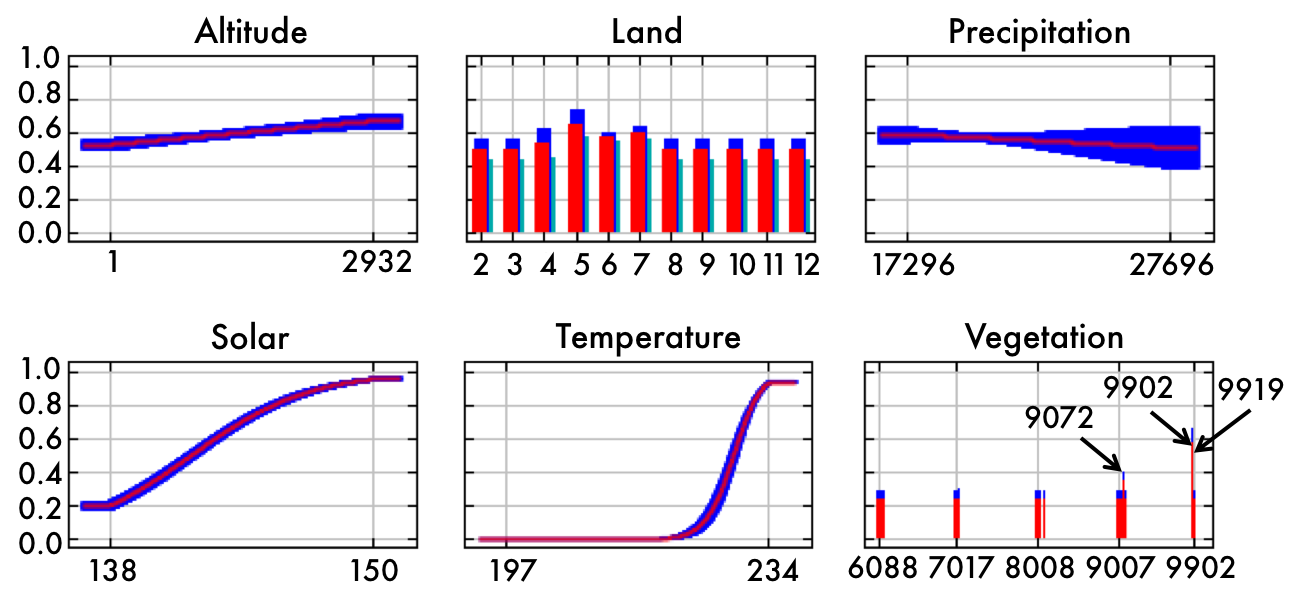


**Figure S4.** Response curves for the prediction based on *A. carolinensis* presence data of Okinawa populations. Land = land-use, and Solar = global solar irradiance. Units: altitude, 0.1m; precipitation, 0.1 mm; solar, 0.1 MJ/m^2^; temperature, 0.1°C. Land-use and vegetation types are categorical variables. Land-use types: 2, body of seawater; 3, wasteland; 4, forest; 5, other agricultural land; 6, land for building; 7, other land; 8, beach; 9, golf course; 10, rivers and lakes; 11, trunk transportation land; 12, paddy field. Vegetation types: 9072, farmland weed community; 9902, urban area; 9919, artificial land. Red color represents mean whereas blue color represents one standard deviation.


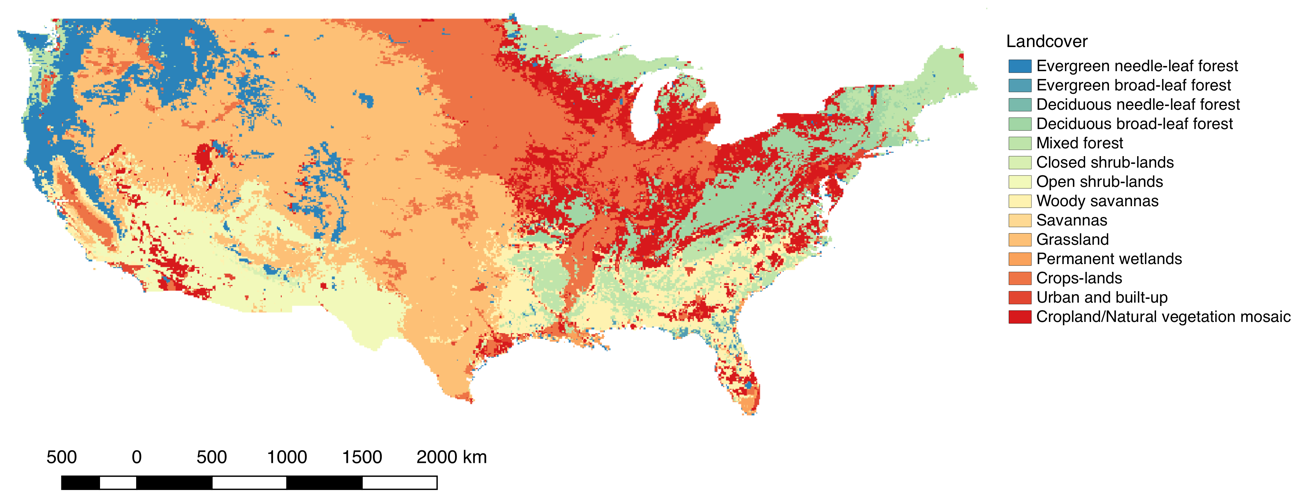


**Figure S5.** Land cover map of North America. Light blue indicates evergreen broad-leaf forest.
